# Role for autophagy-related markers Beclin-1 and LC3 in endometriosis

**DOI:** 10.1186/s12905-022-01850-7

**Published:** 2022-06-29

**Authors:** Zhiwei Kong, Tingting Yao

**Affiliations:** 1 Department of Gynecological Oncology, Sun Yat-Sen Memorial Hospital, Sun Yat-Sen University, 107 Yan Jiang West Road, Guangzhou, 510120 People’s Republic of China; 2 Key Laboratory of Malignant Tumor Gene Regulation and Target Therapy of Guangdong Higher Education Institutes, Sun Yat-Sen University, Guangzhou, China; 3 Department of Gynecological Oncology, Nanhai Maternal and Child Heath Hospital, Guiping West Road, Guicheng, Nanhai District, Foshan, 528200 China

**Keywords:** Endometriosis, Autophagy, Beclin-1, LC3, Immunohistochemistry

## Abstract

**Background:**

Endometriosis is a common and challenging disease in women of childbearing age with high personal and social costs. Many molecular differences between ectopic and eutopic endometrium present difficulties in the development of new drug therapies and therapies. Autophagy is a response to stress and has recently been studied in human cancers. Two important autophagy genes, Beclin-1 and LC3, have been reported in several human cancers. However, the reports of Beclin-1 and LC3 in endometriosis are limited and controversial.

**Methods:**

In this study, we investigated the expression of Beclin-1 and LC3 by Immunohistochemistry.

**Results:**

We found their downregulation in endometriosis. We also found these two autophagy gene expression are negatively correlated with the stage of endometriosis.

**Conclusions:**

Decreased expression of Beclin-1 and LC3 may be related to the occurrence and development of endometriosis.

## Background

Endometriosis is a common, chronic inflammatory disease characterized by hormone-dependent endometrial cells including glandular cells and stromal cells grow in ectopic sites. It is reported that endometriosis affected approximately 10% of women worldwide, and 50–60% of these women got pelvic pain [1]. Moreover, up to 50% of female infertility is caused by endometriosis. Treatment for each endometriosis patient cost nearly $12,000 [2], while mechanism still remains unclear.

Autophagy is an evolutionarily conserved and highly regulated lysosomal degradative pathway that degrades macromolecules and organelles inside of the cell. Autophagosome formation and maturation are regulated by several core autophagy gene (*Atg*) proteins in a highly controlled manner. Thirty specific genes have been identified in yeast that regulate autophagy, sixteen are homologous to humans [3]. Beclin-1 with a molecular weight of approximately 60 kDa is located on chromosome 17q21. Itis a mammalian cell homolog of the yeast autophagy-related gene *Atg6*, and mediates the initiation stage of autophagy [4].

Microtubule-associated protein light chain 3 (LC3) is another key protein in autophagy. It is encoded by a mammalian ortholog of yeast *Atg8* to produce a soluble protein (approximately 17 kDa) that is ubiquitous in mammalian tissues and cultured cells. LC3 has two forms, LC3-I and LC3II.Both forms play major roles in cytoplasm of autophagosomes and autologous lysosomes [5]. Studies on autophagy and endometriosis are rarely reported, and the results are inconsistent. One study showed that autophagy promoted the transition of endometrial epithelial-mesenchymal cells, and that LC3 was elevated in ectopic endometrium contributing to the development of endometriosis [6]. Conversely, another study showed that LC3 was downregulated in the ectopic endometrium and autophagy could inhibit endometriosis, [7].

In our study, immunohistochemistry was used to detect expression of Beclin-1 and LC3 in ectopic endometrial tissues and normal endometrium.

## Methods

### Sample collection

84 women diagnosed with endometriosis received surgery in Gynecology Department of Nanhai District Maternal and Child Health Hospital of Foshan City from November 2016 to October 2018 (Table 1) were provided informed consent. This study was approved by the Ethics Committee of this hospital. The patients ranged from 26 to 49 years (average, 34 ± 6.9 years) had received laparoscopic ovarian endometriosis cystectomy.

**Table 1:** Clinical data for the 84 experimental group patients with endometriosis

Project	Number of cases (n)	Percentage
*Age (years)*		
≤ 33	55	0.655
> 33	29	0.345
*Staging*		
Phase I (0–5 points)	1	0.012
Phase II (6–15 points)	31	0.369
Phase III (16–40 poiSnts)	19	0.226
Phase IV (> 40points)	33	0.392

The control group comprised 32 patients, aged 22–48 years (average, 39.2 ± 7.0 years) from the same hospital who underwent endometrial curettage or total hysterectomy because of tubal infertility and uterine fibroids. All of patients underwent surgery in the proliferative phase of the menstrual cycle, following 3–7 days after menstruation. None took steroids, or in pregnant, or in lactation 6 months before any of the surgeries.

All of them were inquired for a detailed medical history, including of vaccinations, menstruation, marriages, and family. All received a detailed physical examination, laboratory and functional examinations, including hematuria, coagulation, vaginal secretions, liver and kidney function, thyroid function, electrocardiogram, detailed pelvic abdominal B ultrasound, and chest X-ray, to rule out the presence of any other endocrine diseases.

### Immunohistochemistry

Paraffin-embedded tissues were archived. Each block was serially sectioned at 4 µm thickness for immunohistochemical staining. Baked at 60 °C for 2 h, deparaffinized and rehydrated for high-pressure, heat-mediated antigen retrieval for 5 min. The sheets were blocked endogenous peroxidase activity by using 3% hydrogen peroxide in 10 min, followed by washes (5 min each) with 0.01 M phosphate-buffered saline (PBS), and then blocked with the same serum as the secondary antibody source for 20 min at room temperature. The working solution for the primary detection antibodies, rabbit anti-Beclin-1(Abcam, ab55878, Cambridge, UK) and rabbit anti-LC3A/B (Abcam, ab48394, Cambridge, UK), was prepared in accordance with the primary antibody specification. The primary detection antibodies and the tablets were placed in a wet box and transferred to a 4 °C refrigerator overnight, then washed three times with 0.01 M PBS (5 min each). Using the instructions for the horse radish peroxidase-labeled secondary antibody, the concentration of the secondary antibody was determined, and incubated with the homologous primary antibody in the wet box for 1 h at room temperature. The sheets were washed three times with 0.01 M PBS, incubated with streptavidin–biotin complex in a wet box for 30 min at room temperature, and washed another three times with 0.01 M PBS (5 min each). Immuno-labeling was visualized by dropwise addition of 3,3′-diaminobenzidine color staining solution for 5 to 10 min. The sheets were washed with water, and the tablets were stained with hematoxylin, then dehydrated and finally sealed with a neutral gum.

The sections were independently reviewed by two pathologists using a double-blind method. Beclin-1 was mainly expressed in the membrane and cytoplasm. LC3 was mainly expressed in the cytoplasm. Some nuclei and stroma were visible when strongly positive.

We observed five 40 × 10 high power fields at random; 100 cells were counted, and the color depth of the cells was observed. Ultimately, the ratio of cells and the depth of staining were assessed comprehensively. Beclin-1 and LC3 have the same scoring criteria.

1. Positive staining cell score: 1, positively stained cells accounting for ≤ 10% of the total cells; 2, positively stained cells accounting for 11–50% of the total cells; and 3, positively stained cells accounting for 51%–75% of the total cells.
2. Dyeing depth score: 0, no staining; 1, light yellow; 2, yellow; and 3, dark yellow to brown.
3. Total score: positive staining cell ratio score × staining intensity score; ≤ 3 is negative, > 3 is positive.

### Grading and staging of endometriosis

The original records and surgical images of the endometriosis group were reviewed. Using the records of the location, number, size and adhesion of the endometriotic lesions, the American Society for Reproductive Medicine classification was used to correct the endometriosis staging method [3].

### Statistical analysis

Statistical analysis was performed using SPSS 22.0 software. Expression of Beclin-1 and LC3 protein in both groups, and in subgroups at different stages of endometriosis, were analyzed by the chi-square test. Spearman correlation analysis was used to analyze the correlation. *P* < 0.05 was used to indicate a statistically significant difference.

## Results

### Expression of Beclin-1 and LC3 Immunohistochemistry

**Fig. 1:**
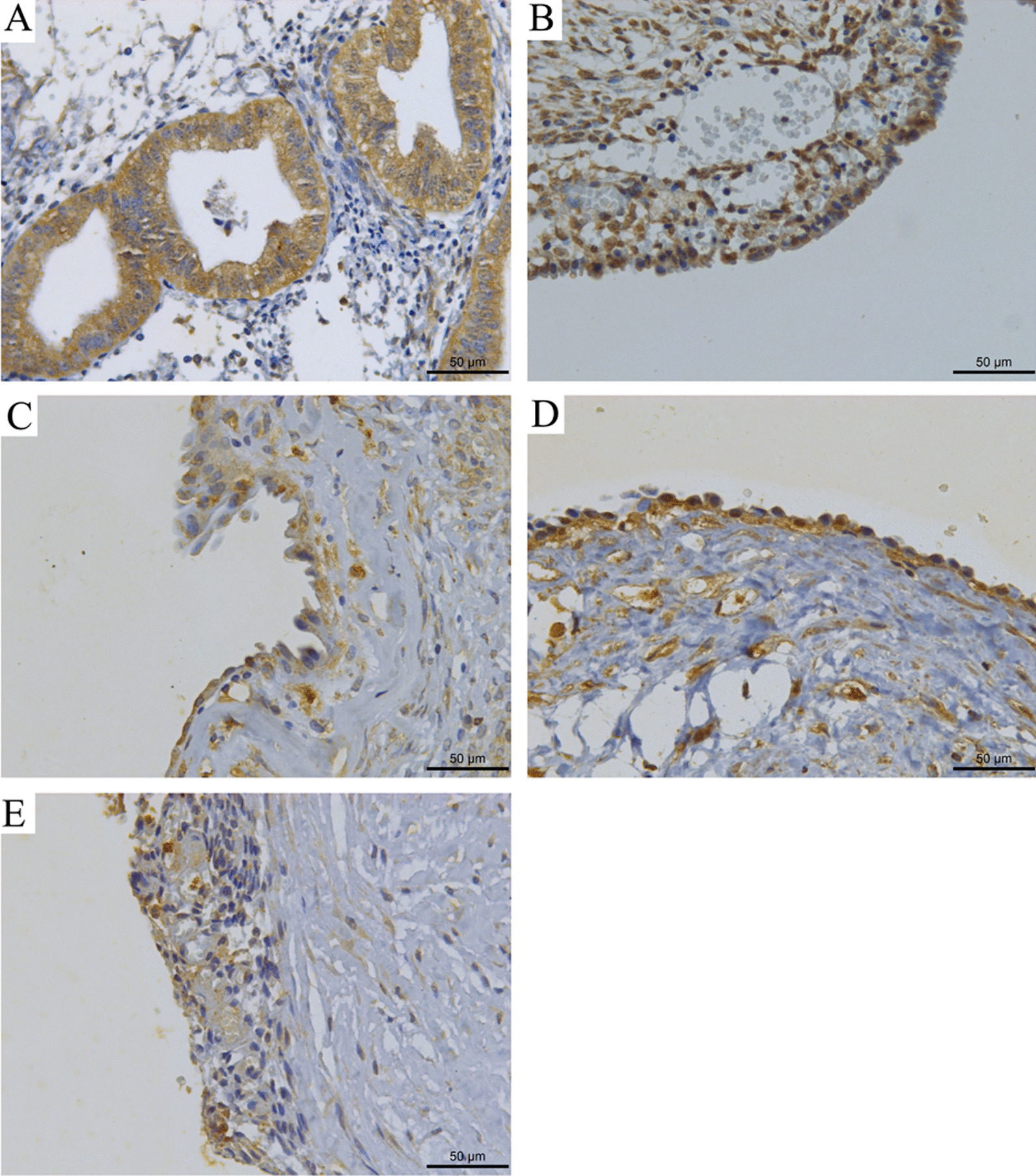
Expression of Beclin-1 in normal endometrium and ectopic endometrium **A** Expression in normal endometrial tissue. **B** Expression in phase I endometriosis. **C** Expression in phase II endometriosis. **D** Expression in phase III endometriosis. **E** Expression in phase IV endometriosis

**Fig. 2:**
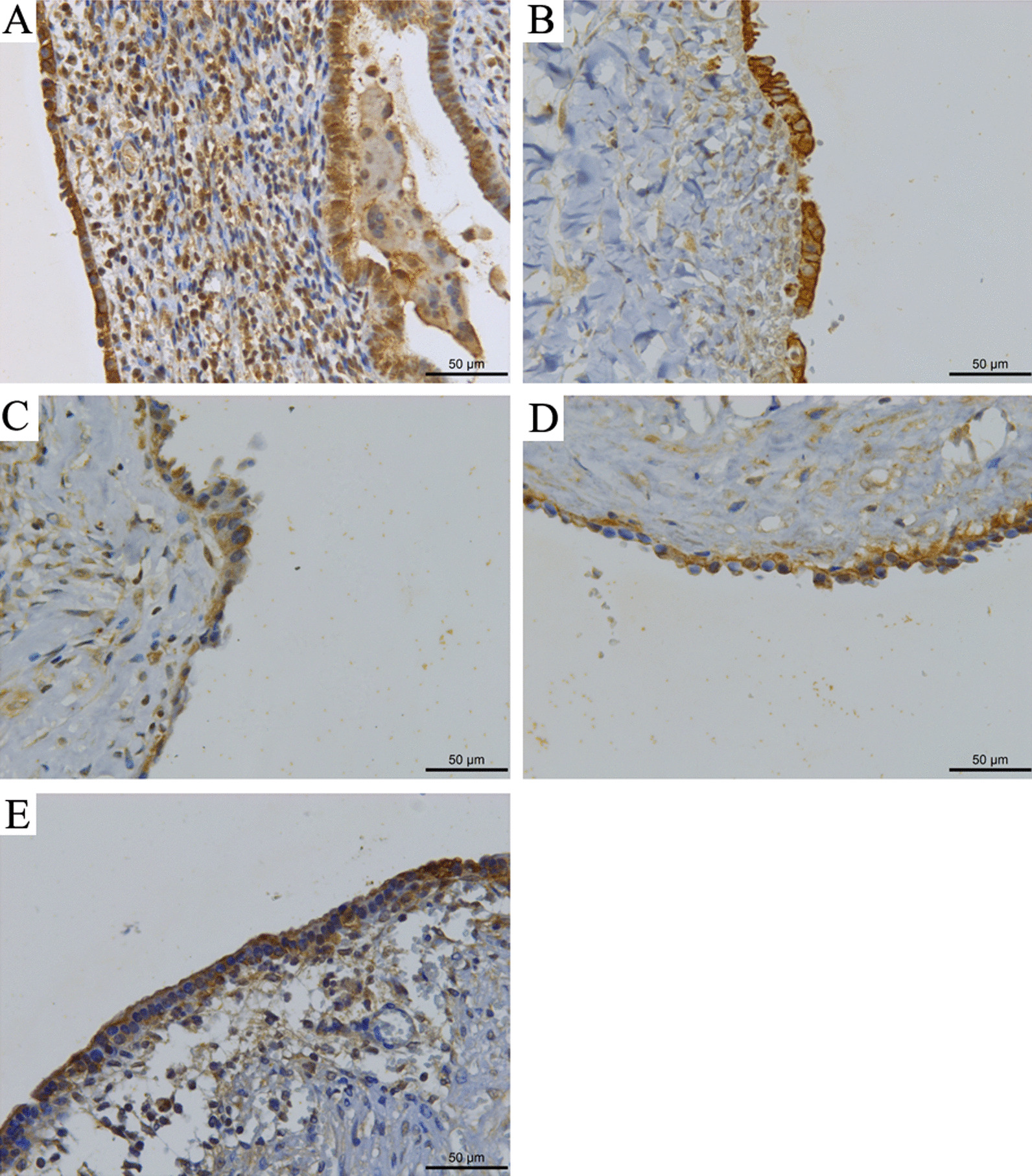
Expression of LC3 in normal endometrium and ectopic endometrium. **A** Expression in normal endometrial tissue. **B** Expression in phase I endometriosis. **C** Expression in phase II endometriosis. **D** Expression in phase III endometriosis. **E** Expression in phase IV endometriosis

Beclin-1 protein was detected in both groups (Fig. 1A–E); however, the rate of positivity in endometriosis group (67.9%; 62/84), was significantly lower than that in control group (96.9%; 31/32; *P* < 0.05). LC3 was mainly expressed in cytoplasm, and was also expressed in some cell stroma and nuclei (Fig. 2). The rate of positivity in endometriosis group was 76.1% (64/84), which was significantly lower than control group (93.75%; 30/32; *P* < 0.05) (Table 2).

**Table 2:** Expression of Beclin-1 and LC3 in the experimental and control groups

Group	Beclin-1 (n)				LC3 (n)			
	Positive	Negative	*X*^*2*^	*P*	Positive	Negative	*X*^*2*^	*P*
Experimental Group	62	22	10.62	0.007	64	20	6.81	0.009
Control Group	31	1			30	32		

### Relationship between Beclin1 and LC3 and clinical stage of endometriosis

For subgroup analysis, because of the small sample size, the single patient in stage I endometriosis (score, 4 points), was combined with the phase II patients for analysis. There were 32 cases of stage I and II endometriosis in the endometriosis group, and the rate of Beclin-1 positivity was 93.8% (30/32). There were 19 cases of stage III and 33 cases of stage IV endometriosis, with rates of Beclin-1 positivity of 68.4% (13/19) and 57.6% (19/33), respectively. The difference was statistically significant (Table 3).

**Table 3:** Expression of Beclin-1 protein in patients with different stages of endometriosis

Staging	N	Negative	Positive	*X*^*2*^	*P*
Phase I–II (1–15 points)	32	2	30	11.37	0.003
Phase III (16–40 points)	19	6	13		
Phase IV (> 40 points)	33	15	19		

The rate of LC3 positivity in the 32 cases of stage I and II endometriosis was 96.9% (31/32), 78.9% (15/19) in the 19 cases of stage III, and 54.5% (18/33) in the 33 cases of stage IV endometriosis. The difference was statistically significant (Table 4).

**Table 4:** Expression of LC3 in patients with different stages of endometriosis

Staging	N	Negative	Positive	*X*^*2*^	*P*
Phase I–II (1–15 points)	32	1	30	16.15	0
Phase III (16–40 points)	19	4	15		
Phase IV (> 40 points)	33	15	18		

The chi-square segmentation method was used to analyze differences in autophagy-associated markers between stage III and stage IV endometriosis. The results showed that there was no significant difference for Beclin-1 expression or LC3 expression between stage III and stage IV. Therefore, these two sets of data were combined and compared with those for stage I and stage II.

Among stage III and IV endometriosis, Beclin-1 positivity was 61.5%. LC3 positivity in the stage III and IV was 63.5%, a statistically significant difference (Table 5).

**Table 5:** Comparison of Beclin-1 and LC3 protein expression in the subgroups stratified by endometriosis stage

Endometriosis	Beclin-1 (n)				LC3 (n)			
Group	Negative	Positive	*X*^*2*^	*P*	Negative	Positive	*X*^*2*^	*P*
I–II	2	30	10.63	0.001	1	31	12.19	0
III–IV	20	32			19	33		

### Correlation analysis of Beclin-1 and LC3 expression

In the endometriosis group, there were 59 cases of Beclin-1 and LC3 co-positivity, 17 cases of co-negativity, 3 cases of Beclin-1 positivity and LC3 negativity, and 5 cases of Beclin-1 negativity and LC3 positivity. Spearman correlation analysis showed a positive correlation for the expression of both markers in the ectopic endometrium (Table 6).

**Table 6:** Correlation between Beclin-1 and LC3 expression in the experimental group

*LC3*	Beclin-1 (n)		*R*	*P*
	Positive	Negative		
Positive	59	5	0.748	< 0.000
Negative	3	17		

## Discussion

Endometriosis contains ovarian endometriomas, superficial peritoneal implants, and deep pelvic endometriosis. Deep pelvic endometriosis is classified as endometriotic lesions penetrating into the retroperitoneal space or the wall of the pelvic organs to a depth of at least 5 mm [8]. A series of genetic and epigenetic events transmitted at birth can explain endometrial, immunological and placental genetic aspects, susceptibility, and changes associated with endometriosis.

Primordial cells can be endometrial cells, stem cells, or bone marrow cells with genetic and epigenetic defects. These defects, along with other acquired defects that do not manifest, constitute susceptibility. After implantation or metaplasia, defined as stable and disseminated changes, subtle and microscopic lesions appear. These cells require additional genetic or epigenetic changes to alter behavior and develop typical, cystic, deep, or other lesions [9].

Available pharmacological treatments for symptomatic endometriosis work by inhibiting ovulation, lowering serum estradiol levels, and suppressing uterine blood flow. For this reason, there are various pain relief options with similar effects, regardless of the mechanism of action.However,there are still a lot unknown for endometriosis.Such as ineffective immune responses appear to play a key role in the pathogenesis of endometriosis, and there is some scientific evidence that immune responses may be regulated by the microbiome [10].

It has been reported itestinal permeability is abnormal in endometriosis patients, and it might play a role in the pathogenesis of this chronic disease [11]. It has also reported ^1^H-NMR was used to explore metabolic alteration in a cohort of patients with endometriosis which provided new bases for a better understanding of the pathophysiological mechanisms of the disease and for the discovery of new biomarkers [12].

Autophagy is a homeostasis process involving self-digestion for energy. It plays a negligible role in development and differentiation that involve in cancer, infectious diseases, metabolic disease, aging, and neurodegenerative disease [13–17].

Beclin-1 is necessary for formation of autophagosomes. Dysregulation of it, will cause autophagosomes malformation [18]. Beclin-1 has been proved to participate in multiple physiological and pathological processes [19]. Ren has previously revealed downregulation of Beclin-1 in both eutopic endometrium and foci of adenomyosis [20], which were in accordance with ours.

These results indicate that reduction of Beclin-1 leads to autophagy- dependent proteins depression, organelles degradation, and autophagosome formation. Ectopic endometrial cells are then able to escape from autophagy, adhere to the extracellular matrix, invade into tissues, and grow on the outside of endocardium, thus causing endometriosis. Decreasement of mature autophagosomes followed with reduction of Beclin-1 could influence apoptosis of normal cells and lead to the survival of ectopic endometrium. Inactivation of Beclin-1 cause the loss of regulatory control of Bcl-2 which may stop autophagy reflux and the programmed cell death, thereby promote the survival of ectopic endometrial cells. The deposition of ectopic endometrium stimulates the activation of local and general inflammation, including increases in chemokines and other pro-inflammatory cytokines, which can accelerate the occurrence of endometriosis.

LC3 is mainly localized in pre-autophagic vacuoles and at the membrane surface of autophagic vacuoles. It’s active constituent is lipo-LC3-II, with the effect of being selectively incorporated into the autophagic substrate and enhancing coupling of the expansion and closure of the autophagic membrane [20]. LC3 is also capable of giant cell ingestion and antigen presentation [21]. Our research has shown that the rate of LC3 positivity was reduced compared with that in the control group. We suggest ectopic endometrial cells produce less autophagosmes or smaller autophagosmes, thus diminishing the efficiency and function of autophagosome formation, reducing the exercise ability of autophagy, failing to combine with lysosomes, decreasing the formation of autophagic lysosomes and autophagic cell death, which make the ectopic endometrial cells to escape autophagy and grow ectopically. The reduction of LC3 depresses the ability to digest ectopic endometrial cells, debris and residue; this failure to eliminate the tissue triggers inflammatory and immune mechanisms, resulting in local and systematic stimulation thereby activating inflammation. Previous research has demonstrated that interleukin (IL)-8 can stimulate the proliferation of ectopic endometrial cells and enhance endometrial cell adherence to fibronectin, thus promoting the incidence of endometriosis [22].

Our research indicates that expression of LC3 and Beclin-1 decreases with increasing clinical stages of endometriosis. Furthermore, the positive correlation between them suggests reduced autophagic activity of ectopic endometrial cells may induce endometriosis. The ability to digest ectopic endometrial cells through autophagic mechanisms is depressed and, because of local inflammatory infiltration and dysimmunity, ectopic endometrial cells exhibit a stronger capacity for angiogenesis. Proliferation and migration of ectopic endometrial cells activated by angiogenic factors could mutually promote both the occurrence and development of endometriosis, in a manner similar to colorectal cancer [23].However, our research chose just one method for detection and just reflected protein level. So we will detect autophagy mechanism deeply.

## Conclusions

Beclin-1 and LC3 are downregulated in endometriosis and negatively correlated with clinical stage of endometriosis, and might be involved in the occurrence and development of endometriosis. Interactions between these two may boost the development of endometriosis. Further research should combine them with human epididymis protein 4 and cancer antigen 125 to scrutinize the effect of autophagy on endometriosis. It would also be useful to explore the relationship between ovarian hormones and autophagy in endometriosis. Autophagy regulation could become a new approach in the diagnosis and treatment of endometriosis.

## Data Availability

The datasets used and/or analysed during the current study are available from the corresponding author on reasonable request.

## Abbreviations

*Atg*: Autophagy gene
LC3: Light chain 3
IL: Interleukin
